# Adoption of Clean Cookstoves after Improved Solid Fuel Stove Programme Exposure: A Cross-Sectional Study in Three Peruvian Andean Regions

**DOI:** 10.3390/ijerph14070745

**Published:** 2017-07-08

**Authors:** Jennyfer Wolf, Daniel Mäusezahl, Hector Verastegui, Stella M. Hartinger

**Affiliations:** 1 Swiss Tropical and Public Health Institute, 4051 Basel, Switzerland; jennyfer.wolf@unibas.ch (J.W.); stella.hartinger.p@upch.pe (S.M.H.); 2 Centre for African Studies, University of Basel, Petersplatz 1, 4001 Basel, Switzerland; 3 Universidad Peruana Cayetano Heredia, Av. Honorio Delgado 430, Urb. Ingeniería, S.M.P., 31 Lima, Peru; hector.verastegui.h@upch.pe

**Keywords:** clean cookstoves, adoption, stove stacking, Peru, household air pollution, improved solid fuel stove, liquefied petroleum gas

## Abstract

This study examined measures of clean cookstove adoption after improved solid fuel stove programmes in three geographically and culturally diverse rural Andean settings and explored factors associated with these measures. A questionnaire was administered to 1200 households on stove use and cooking behaviours including previously defined factors associated with clean cookstove adoption. Logistic multivariable regressions with 16 pre-specified explanatory variables were performed for three outcomes; (1) daily improved solid fuel stove use, (2) use of liquefied petroleum gas stove and (3) traditional stove displacement. Eighty-seven percent of households reported daily improved solid fuel stove use, 51% liquefied petroleum gas stove use and 66% no longer used the traditional cookstove. Variables associated with one or more of the three outcomes are: education, age and civil status of the reporting female, household wealth and size, region, encounters of problems with the improved solid fuel stove, knowledge of somebody able to build an improved solid fuel stove, whether stove parts are obtainable in the community, and subsidy schemes. We conclude that to be successful, improved solid fuel stove programmes need to consider (1) existing household characteristics, (2) the household’s need for ready access to maintenance and repair, and (3) improved knowledge at the community level.

## 1. Introduction

Nearly three billion people continue to use solid fuels for cooking, heating and lighting [1]. Exposure to solid fuel smoke often through the use of traditional cookstoves (“traditional cookstove” includes open indoor fire in this article) is associated with adverse health impacts like respiratory infections [2,3], ischemic heart disease [4], stroke [5], lung cancer [6], and cataract formation [5,7]. Household air pollution was ranked the eighth leading health risk factor and was associated with nearly three million deaths in 2015 [8]. The use of traditional cookstoves has furthermore been shown to lead to negative social and environmental impacts through excess time and money spent [9], contribution to outdoor air pollution [10,11], deforestation and climate change [12].

To mitigate the negative impacts of burning solid fuels for cooking, efforts to promote the adoption of clean cookstoves are undertaken. The Global Alliance for Clean Cookstoves was created to foster household adoption of clean cookstoves and fuels [13]. Additionally, one of the Sustainable Development Goals calls for access to affordable, reliable, sustainable and modern energy for all [14]. However, programmes promoting clean cookstoves frequently experienced problems; households did not sufficiently or sustainably use the new stove, nor did maintain or repair it when broken [15,16]. Near-exclusive use of clean cookstoves is essential to sufficiently reduce air pollution levels to achieve positive health impacts [17,18]. Often, however, clean cookstoves are just added to the traditional stoves leading to a parallel use of cooking devices (“stove stacking”) [19].

Research on clean cookstove adoption has discovered enablers and barriers for clean cookstove adoption. Puzzolo et al. [20] identified factors related to the cooking technology, the household, community, programme and societal level. A Chinese literature review identified stove design, knowledge and awareness, household characteristics, market development, financial support and favourable policies as important enablers for clean cookstove and fuel adoption [21]. Household wealth and education, demographics, stove programme characteristics, and costs were described as important factors across settings [22,23,24,25,26].

Evaluation of promotion programmes needs to assess whether households use and value the new stove. An additional important measure is the displacement of traditional stoves when clean cookstoves are introduced [17]. Knowledge of enabling and hindering factors is essential for programme monitoring and future efforts. Here we present results from 1200 questionnaires administered to rural Andean households that had previously taken part in an improved solid fuel stove (IS) promotion programme. The objectives of this study were to explore adoption and displacement of different stove types as well as associated factors in diverse rural Andean regions where the use of solid fuels is widespread.

## 2. Materials and Methods

### 2.1. Study Settings

The study was conducted between February and November 2014 in three rural Andean Peruvian regions: Cajamarca, Cuzco and La Libertad. In rural Peru, wood is the most important cooking fuel and is exclusively used by over 40% of households. Many more use wood in combination with other solid or with clean fuels such as liquefied petroleum gas (LPG). LPG is used exclusively by only 5% of the rural population [27]. The study sites were selected purposively because of (i) large IS promotion programmes which had been conducted within the last 15 years, (ii) geographical and cultural differences between the regions, (iii) knowledge of the study sites and availability of data on e.g., the number of households exposed to IS promotion programmes, and (iv) a sufficiently high number of households in the communities. More information on the study sites can be found in the Supplementary Material (Text S1).

### 2.2. Sampling, Inclusion Criteria and Selection of Households

For the study site selection we identified the number of IS promotion programmes in the province, districts and communities of the area from local non-governmental organisations (NGOs), municipal and community authorities. We then sampled households from available census lists from all communities in the study sites. A household was considered eligible for the study (i) if it had previously taken part in an IS promotion programme of a governmental, non-governmental or research institution, (ii) if it used biomass (wood, carbon, dung, etc.) for cooking (alone or in combination), (iii) if it spoke Spanish as first language, and (iv) if its household head was at least 18 years old. From all eligible households, a convenience sample was drawn; households in a distance perimeter of a one hour car’s drive from the community centre and accessible within a maximum of 20 min walk from a main road were visited. Only households willing to participate were interviewed. Data collection on stove use and potentially explanatory variables was performed as described in the section below.

### 2.3. Study Design and Data Collection

The questionnaire for this cross-sectional study was informed by recent literature on indices of clean cookstove adoption [28] and their enablers and barriers [20,21,22,23,24,25,26]. The questionnaire was pre-tested by our field staff before the start of the study. Where there were doubts about the comprehensibility of questions or related answers, changes were applied immediately and re-checked. After having completed pre-testing, we piloted the questionnaire in 20 households in San Marcos, Cajamarca. Additional modifications were made as needed. The final questionnaire took approximately two hours to complete. It included both open and closed questions and was written in Spanish. The main topics included clean cookstove adoption, i.e., questions on cooking behaviours, types of cookstoves present in the household, patterns of cookstove use, cleaning, maintenance and repair, willingness to pay for an IS or its parts and fieldworker observations and demographic, socio-economic and livelihood variables. All interviewers received at least three days of specific training on the questionnaire and performed practice interviews before commencing the study. A questionnaire-based interview was conducted with the female household head and field supervisors reviewed each questionnaire after completion for errors or missing values. More information on the questionnaire can be obtained from the corresponding author.

We also conducted a total of 25 focus group discussions (FGDs) in the three regions to explore, for example, general perceptions of the different stoves and barriers and enablers for their adoption. Results of FGDs will be presented elsewhere.

### 2.4. Data Analysis

We used multivariable logistic regression models to examine three outcomes of interest: (a) reported daily IS use, (b) reported LPG stove use, and (c) reported displacement of the traditional stove (i.e., the traditional cookstove was no longer used). Sixteen independent variables were chosen a priori to be included in the regression models. These variables were chosen based on a review of the literature and included (i) demographics and household variables (region, age, education and civil status of reporting female, household size, household wealth index, land ownership (where the house is built)), (ii) knowledge and awareness variables (education and internet use), (iii) price and costing variables (household wealth index, firewood needs to be bought, stove programme, taking part in national gas programme), (iv) stove design variables (stove programme, problems with IS), variables describing the supply chain, the clean cookstove market and an enabling environment (knowledge of somebody who can build an IS, IS parts can be obtained in the community, local authorities/leaders support IS use, taking part in the national gas programme). Variables, their categorisation and missing values are listed in Table S1. Education was categorised as zero years, one to six years—which corresponds to primary school—and above six years of schooling. All 16 explanatory variables were included in all three regression models independent of their *p*-value or the effect on the estimate of one of the other variables. Effect estimates presented in this paper are therefore adjusted for all other variables in the model.

Complete information was available for 87% of all participating households, i.e., for 13% of households there was at least one of the independent variables for logistic regression analyses missing (Table S1). Multiple imputation using chained equations was performed to impute missing data. More details can be found in the Supplementary Material (Text S2 and Tables S2–S5). Results of the logistic regression models presented in this article are based on the imputed dataset.

Analyses were performed using Stata/IC 14 (StataCorp, 2015, Stata Statistical Software, College Station, TX, USA).

### 2.5. Ethics

The study was approved by the ethical review boards of the Universidad Peruana Cayetano Heredia in Lima, Peru, and the University of Basel, Switzerland (through the ethics commission ‘Ethikkommission Nordwest- und Zentralschweiz’ (EKNZ)). All household heads signed written informed consent prior to participation.

## 3. Results

In the three regions, Cajamarca, Cuzco and La Libertad, stove use data and data on potentially explanatory variables were collected from 48, 12 and 23 communities, respectively. In total, 1202 households participated and 400 questionnaires were administered in Cajamarca, 400 in Cuzco and 402 in La Libertad. We excluded 169 households or 14% of all data from the analysis. Of those, 99 households were excluded because they had not taken part in a stove programme, but had adopted their own IS following experiences with cooking on their neighbour’s or friend’s IS. The remainder were excluded because of implausible values in the stove use variables. Demographics and further information on the 1033 remaining households are given in Table 1.

### 3.1. Stove Use and Adoption

Numbers and percentages of households using clean and traditional stoves as the primary or secondary cookstove, using the IS daily, using the LPG stove and having displaced the traditional stove are shown in Table 1. Some 87% of women stated that they used the IS daily, 51% that they used the LPG stove (in general) and 66% that they did not use traditional cookstoves. Ninety-two percent of households stated that they used clean cookstoves as the primary cooking technology, with the IS being the most important reported primary cookstove. In case a secondary cookstove was mentioned, this was mostly an LPG stove. Fieldworker observations on households reporting the IS as the primary or secondary stove revealed a missing or broken chimney in 3.5% and 6.1% and no closed combustion chamber in 13% of all observations. Eighteen percent of all households stated that they participated in the national gas programme “Fondo de Inclusión Social Energético” (FISE) [30], 69% practised stove stacking (i.e., mentioned using a secondary stove) and 54% and 79% were willing to pay (any amount of money) for a new IS or replacement parts in case the current stove would break.

### 3.2. Multivariable Analysis of Factors Associated with Clean Cookstove Adoption

Results of the multivariable logistic regression models examining daily IS use, use of the LPG stove and traditional stove displacement are presented in Table 2. Taking part in the national gas programme FISE [30] was an almost perfect predictor of being a gas user. This variable was therefore not included in the regression model but analysis of gas use was performed including only those households that had not taken part in the national FISE programme (*n* = 844). In the following paragraphs, we list the explanatory variables that are associated with one of the three outcomes which we define as a *p*-value of ≤0.05. If a variable consists of several categories, such as education, we refer to the overall *p*-value as given in Table 2.

#### 3.2.1. Daily IS Use

Variables associated with daily IS use in the multivariable logistic regression analysis were years of schooling and civil status of the reporting female (odds ratios, ORs,) and *p*-values of all variables are given in Table 2, second column). Encountering problems with the IS was negatively associated (OR 0.62 (0.41, 0.93)) and knowledge of somebody able to build or repair an IS was positively associated with daily IS use (OR 2.01 (1.24, 3.27)).

#### 3.2.2. LPG Stove Use

Variables associated with LPG stove use in the multivariable logistic regression analysis were years of schooling (e.g., OR 2.47 (1.26, 4.87) for ≥7 years compared to no schooling) and wealth index (e.g., OR 10.17 (5.67, 18.22) for the fifth quintile compared to the first quintile). Knowledge of somebody able to build an IS was negatively associated with LPG stove use (OR 0.68 (0.47, 0.98)) (Table 2, third column for all ORs and *p*-values).

#### 3.2.3. Traditional Stove Displacement

Variables associated with the displacement of the traditional stove in the multivariable logistic regression analysis were region, age of the reporting female, household size and wealth index. Encountering problems with the IS was negatively associated (OR 0.65 (0.48, 0.88)) and the possibility to obtain IS parts in the community was positively associated with traditional stove displacement (OR 1.48 (1, 2.2)) (Table 2, fourth column for all ORs and *p*-values).

Tables comparing results from analysis of imputed data and complete cases are shown in Tables S3–S5.

## 4. Discussion

In this study, more than 1000 households had participated in various IS promotion programmes in geographically and culturally diverse rural Andean regions. We show that a large proportion of households report daily clean cookstove use as their main or even as their exclusively used technology, i.e., no concurrent use of traditional cookstoves. Various characteristics are associated with daily IS use, LPG stove use and displacement of the traditional cookstove.

### 4.1. Stove Use and Adoption

Households in our study reported that the IS is their primary stove, however, LPG stoves play an increasing role in household cooking. In a similar setting in Mexico, 50–70% of households continued to use the IS ten months after intervention implementation and only 10% did not adopt it [31]. Usage of the IS was shown to decline during the first months after implementation and then to stabilise [16,31]. In our study in which most households participated in a stove programme several years ago (Table 1), we believe that this stable phase of IS use had been reached. The parallel use of multiple cookstoves (or stove stacking) is common (69% of households). Eighty-five percent of those households combine the IS as the primary stove with either the LPG (63%) or the traditional stove (22%). These numbers vary by region: in Cuzco 76% of all households practicing stove stacking use the IS as the primary plus the LPG stove as the secondary stove, whereas in Cajamarca this number is only 50% and more households (33% of all households practicing stove stacking) combine the IS with a traditional stove. Exclusive IS use is a rare phenomenon, as clean cookstoves seldom meet all household needs [19,32]. However, 66% of households reported not using the traditional cookstove, but using either the IS exclusively or in combination with the LPG stove. During our previous research activity in Cajamarca in 2009 [33,34], household use of LPG was still uncommon. However, having accepted the IS as new cooking technology might have increased households’ acceptance of other clean stoves, a phenomenon also described in Mexico [31]. From focus group discussions that we conducted within the framework of this study, we discovered that households were highly enthusiastic about LPG stoves. However, these stoves were not considered as true replacements for solid fuel stoves and mostly used for minor cooking activities of short duration (such as breakfast preparation and heating dinner). Reasons given in focus groups were the high price of LPG and alteration in the taste of traditional meals.

All three measures of stove use, i.e., daily IS use, LPG stove use and traditional stove displacement, are important to achieve health effects and to describe different phases after clean cookstove promotion. A household might, for example, use the IS but might not yet have switched to LPG or discontinued using the traditional stove. Indeed, use of the IS or the LPG stove might be a prerequisite for traditional stove displacement. In this sense, traditional stove displacement might reflect sustained changes after clean cookstove promotion. In our study, 70% of households that reported daily IS use, also reported traditional stove displacement, as compared to only 35% in households with no daily IS use. Similarly, 73% of households using the LPG stove reported traditional stove displacement compared to 57% of households not using the LPG stove.

### 4.2. Factors Associated with Cookstove Adoption and Displacement

In-depth understanding of local enablers and barriers has been postulated to be key for successful clean cookstove interventions [35,36]. We selected our list of potential important factors based on prior evidence and explored them in a specific local context. Similar to what has been described before, some of our enabling and hindering factors are relevant for more than one outcome. Variables associated with clean cookstove adoption (i.e., with one of our three outcomes) can be grouped in demographics (education, age, civil status and region), household (household size, household wealth index), community (knowledge of somebody able to build or repair an IS and whether IS parts are obtainable in the community), stove (encounters of problems with the IS) and policy characteristics (subsidy schemes such as the national gas programme FISE). Women with higher education had higher odds of adopting the LPG stove, but lower odds of using the IS daily. Higher education was consistently associated with increased uptake of clean cookstoves in previous research [20,21,23,24]. In the present settings where the IS has become a standard cooking technology (reflected by high usage), LPG stoves might be perceived as new and modern and as a stove that more educated and wealthy households can access initially. Related to that, women in the lowest age group had higher odds of displacing the traditional stove and higher household wealth was associated with both traditional stove displacement and LPG stove use. Higher wealth might be especially important in rural areas where LPG is usually more expensive [20]. Women living with a partner had higher odds of using the IS daily than single, separated, divorced or widowed women which might be due to the fact that these women cook more often and for more people. Similarly, a bigger household size was associated with a decreased chance of traditional stove displacement, which is consistent with previous research showing LPG stove uptake being larger in smaller households [20]. Experiencing problems with the IS decreased the chance that the household was using the IS daily and also that it had displaced the traditional stove. Fieldworker observations reporting no or a broken chimney of the IS were associated with less daily IS use, less use of the LPG stove and less traditional stove displacement (data not shown). Negative experiences with IS durability are likely to reduce motivation for its use and confidence to abolish traditional stoves. Indeed, stove stacking with traditional cookstoves has been described as a strategy to cope with uncertainties regarding income, fuel prices and access to fuels [19]. Problems with the programme stove were also found to be much lower in communities were new stoves were rapidly taken up compared to communities where uptake took longer [31]. Two more factors, knowledge of somebody with the ability to build or repair an IS (positively associated with daily IS use and negatively associated with LPG use) and whether IS parts are obtainable in the community (positively associated with traditional stove displacement), underline the importance of stove functioning and possibilities for repair and maintenance through a functioning supply chain for clean cookstove adoption. Region was also associated with traditional stove displacement with households in La Libertad and in Cuzco, having higher odds of not using traditional stoves which might be related to policy and programme characteristics. Variables associated with the use of the LPG stove were only examined in the group of households not taking part in the national gas programme [30]. All except one of the 189 households that participated in this programme stated that they used the LPG stove, making the FISE a very strong predictor for LPG uptake. The Peruvian government in 2012 initiated the programme FISE to promote household LPG use for example via subsidies. It has been shown before that initial costs of LPG stoves and cylinders and the high price of LPG in general are among the most important barriers for uptake [20,22] with subsidies being an important measure to raise adoption rates [21]. More information on the different stove programmes is provided Texts S3 and S4, Tables S6 and S7.

### 4.3. Limitations

In cross-sectional study designs, there are a number of different biases that might occur such as selection and information bias. In this study, households were selected from areas in which IS promotion programmes had been conducted. From these areas we included all households having participated in a programme (overall less than 5% of households declined). First, households willing to participate in our study might not be representative of all rural Peruvian households who have taken part in a stove programme. They might have been more or less satisfied with the programme or their respective stove and felt the need to express this. Second, households willing to participate in a stove programme might differ from the average rural Andean household. We nevertheless believe that our results are largely generalisable to the rural Peruvian Andean context as we included a large number of households from geographically and culturally diverse regions.

Furthermore, cross-sectional studies cannot establish temporal sequence between exposure and outcome. Knowledge of somebody able to build an IS was associated with daily IS use, but it is possible that because household members used an IS they knew somebody who could build or repair it. Furthermore, our outcomes and exposures are self-reported which might overestimate actual clean cookstove use. A recent study in Rwanda showed differences between observed and reported IS use [37] (54% observed, 78% reported). A similar observation was made in a humanitarian crisis setting in Darfur, Sudan [38]. Another study found similar results in relation to whether stove use was reported or measured [39]. A study in rural Mexico introducing IS reported similar displacement of the open fire (57% compared to 66% displacement of traditional stoves in this study) [19]. Nevertheless, objective assessment of stove use, for example, with stove use monitors might have led to more accurate outcome assessments [38,39], which was, however, not possible for this study. We believe that in this setting the risk of reporting bias might be smaller as questions on cookstove use were less related to the respective IS promotion programme as there were a considerable number of different programmes which were mostly conducted several years ago. This might, on the other hand, have led to recall bias especially on information on the respective stove promotion programmes. The questionnaire had been designed to evaluate IS use and adoption. Therefore, fieldworker observations relate only to the IS. Observations listing for example all present stoves in the home would have been useful to validate reported IS and LPG stove use and displacement of the traditional stove. We could not examine all potential enablers and barriers described previously and also not all variables that had been included in the questionnaire for this purpose. For example, questions around perceptions of time and money savings, benefits for health and safety and cleanliness of the kitchen were answered very uniformly across households. It was described before that households stated high satisfaction with the programme stove which was not necessarily related to real satisfaction [39]. Finally, we did not assess use of a specific cookstove for cooking the main meal or for other household tasks such as heating water or the home.

## 5. Conclusions

In the Peruvian Andes the transition to clean cookstoves appears to be in process after many years of IS and more recent LPG stove promotion. Only one third of all households report traditional cookstove use and only 8% as the primary stove. These households should be considered in future programmes to achieve desired health impacts. Due to household preferences and related monetary costs, LPG stoves are currently unlikely to completely replace solid fuel stoves. However, LPG stoves offer advantages which might make them very suitable secondary stoves, potentially able to further reduce the role of traditional cookstoves if household economic constraints are carefully considered beyond current subsidy schemes. Factors influencing stove adoption are related to stove maintenance and repair at community-level and a functioning supply chain. Preferences for a certain cookstove seem further influenced by just a few demographic, household, community, stove and public policy characteristics that can be considered for programme planning. The results of our study on more than 1000 households from three geographically and culturally diverse rural Andean settings provide an important evaluation of IS promotion programmes and add to the growing evidence on factors promoting or hindering clean cookstove adoption. The study findings assist in designing successful clean cookstove interventions for the future.

## Figures and Tables

**Table 1:** Background and stove adoption information for 1033 households in three regions of Peru.

Demographics and Further Explanatory Variables	*n* (%)
Region	
Cajamarca	344 (33.3)
Cuzco	331 (32.0)
La Libertad	358 (34.7)
Age of reporting female	
<35	245 (23.7)
35–<45	289 (28.0)
45–<55	249 (24.1)
≥55	230 (22.3)
Education of reporting female (years of schooling)	
0	133 (12.9)
1–6	701 (67.9)
≥7	177 (17.1)
Civil status of reporting female (married/civil partnership vs. single/separated/divorced/widowed)	876 (84.8)
Household size, ≥5 persons	473 (45.8)
Wealth index ^a^	
1. Quintile (lowest)	207 (20.0)
2. Quintile	212 (20.5)
3. Quintile	215 (20.8)
4. Quintile	197 (19.1)
5. Quintile (highest)	202 (19.6)
Land ownership (where house is built)	915 (88.6)
Internet use	341 (33.0)
Firewood is bought	362 (35.0)
Stove programme	
IIN	147 (14.2)
Sembrando	191 (18.5)
Juntos	183 (17.7)
Municipalidad	118 (11.4)
NINA	237 (22.9)
others (various, programmes with <5% coverage)	157 (15.2)
Participation in national gas programme	189 (18.3)
Year of stove programme participation	
≤2008	197 (19.1)
2009	161 (15.6)
2010	193 (18.7)
2011	209 (20.2)
2012	105 (10.2)
2013	50 (4.8)
Problems with IS	338 (32.7)
Knowledge of somebody who can build an IS	332 (32.1)
IS parts can be obtained in the community	174 (16.8)
Local authorities/leaders support IS use	435 (42.1)
**Stove use and adoption**	
Primary cookstove	
IS	902 (87.3)
LPG stove	43 (4.2)
Traditional cookstove ^b^	86 (8.3)
Secondary cookstove	
IS	80 (7.7)
LPG stove	469 (45.4)
Traditional cookstove	167 (16.2)
None	317 (30.7)
Daily IS use	900 (87.1)
LPG stove use	529 (51.2)
Traditional stove displacement	677 (65.5)

^a^ The index is constructed from: (i) number of people per room; (ii) presence of consumer durables; (iii) presence of housing characteristics; (iv) flooring material; (v) drinking-water source; (vi) toilet facility. The original index includes presence of LPG/electricity stove and type of cooking fuel, two variables that we have excluded for the wealth index used in this analysis [29]. ^b^ Includes tulpia (open fire) and traditional cookstove with or without chimney. Acronyms: IIN: Instituto de Investigación Nutricional; IS: improved solid fuel stove; LPG: liquefied petroleum gas.

**Table 2:** Results of multivariable logistic regression analysis of daily IS use, LPG stove use and traditional stove displacement, Peru 2014.

	Daily IS Use (*n* = 1033)		LPG Stove Use (*n* = 844)		Traditional Stove Displacement (*n* = 1033)	
	OR (95% CI)	*p*	OR (95% CI)	*p*	OR (95% CI)	*p*
Region		0.3		0.3		<0.0001
Cajamarca	1		1		1	
La Libertad	1.27 (0.44, 3.68)		1.19 (0.48, 2.94)		2.42 (1.19, 4.93)	
Cuzco	2.46 (0.83, 7.29)		1.71 (0.87, 3.36)		4.48 (2.28, 8.79)	
Age of reporting female		0.4		1		0.002
<35	1		1		1	
35–<45	0.75 (0.44, 1.28)		0.89 (0.56, 1.42)		0.59 (0.39, 0.89)	
45–<55	1.26 (0.68, 2.32)		0.94 (0.57, 1.54)		0.43 (0.28, 0.67)	
≥55	0.96 (0.49, 1.87)		0.93 (0.53, 1.63)		0.49 (0.3, 0.81)	
Education of reporting female (years of schooling)		0.02		0.02		0.2
0	1		1		1	
1–6	0.90 (0.46, 1.76)		2.09 (1.22, 3.6)		1.39 (0.89, 2.17)	
≥7	0.44 (0.20, 0.99)		2.47 (1.26, 4.87)		1.03 (0.57, 1.84)	
Civil status of reporting female, married/civil partnership vs. single/separated/divorced/widowed	1.75 (1.06, 2.88)	0.03	0.97 (0.62, 1.54)	0.9	0.98 (0.65, 1.47)	0.9
Household size, ≥5 persons	1.25 (0.82, 1.91)	0.3	0.80 (0.56, 1.13)	0.2	0.64 (0.47, 0.88)	0.005
Wealth index		0.9		<0.0001		0.001
1. Quintile (lowest)	1		1		1	
2. Quintile	1.05 (0.57, 1.93)		2.41 (1.4, 4.15)		1.40 (0.92, 2.14)	
3. Quintile	0.77 (0.43, 1.4)		2.03 (1.17, 3.51)		1.81 (1.17, 2.8)	
4. Quintile	0.93 (0.48, 1.78)		5.05 (2.92, 8.74)		1.27 (0.81, 1.99)	
5. Quintile (highest)	0.91 (0.46, 1.79)		10.17 (5.67, 18.22)		2.74 (1.65, 4.54)	
Land ownership	1.06 (0.58, 1.93)	0.9	0.97 (0.57, 1.65)	0.9	0.77 (0.47, 1.26)	0.3
Internet use	1.36 (0.85, 2.18)	0.2	1.28 (0.89, 1.83)	0.2	1.07 (0.76, 1.5)	0.7
Firewood is bought	0.80 (0.52, 1.23)	0.3	1.09 (0.76, 1.55)	0.6	1.28 (0.92, 1.77)	0.1
Stove programme		0.2		0.07		0.1
IIN	1		1		1	
Sembrando	2.32 (0.74, 7.25)		1.04 (0.44, 2.47)		1.48 (0.73, 2.98)	
Juntos	2.37 (0.68, 8.27)		2.61 (0.99, 6.9)		0.80 (0.36, 1.78)	
Municipalidad	3.21 (0.75, 13.76)		1.13 (0.4, 3.21)		0.66 (0.27, 1.64)	
NINA	1.42 (0.28, 7.32)		1.43 (0.47, 4.34)		0.51 (0.19, 1.39)	
others (various, <5%)	3.91 (1.11, 13.78)		1.39 (0.57, 3.43)		0.93 (0.45, 1.93)	
Year of stove programme participation		0.2		0.1		0.2
≤2008	1		1		1	
2009	0.48 (0.13, 1.86)		0.54 (0.2, 1.46)		0.96 (0.43, 2.14)	
2010	0.33 (0.09, 1.24)		0.90 (0.36, 2.26)		1.00 (0.46, 2.17)	
2011	0.41 (0.11, 1.61)		0.87 (0.34, 2.25)		1.24 (0.53, 2.91)	
2012	0.77 (0.19, 3.22)		1.61 (0.62, 4.16)		1.42 (0.62, 3.25)	
2013	0.31 (0.07, 1.38)		0.98 (0.33, 2.88)		3.23 (1.18, 8.84)	
Problems with IS	0.62 (0.41, 0.93)	0.02	1.13 (0.8, 1.6)	0.5	0.65 (0.48, 0.88)	0.005
Knowledge of somebody who can build IS	2.01 (1.24, 3.27)	0.01	0.68 (0.47, 0.98)	0.04	1.02 (0.74, 1.41)	0.9
IS parts can be obtained in community	0.84 (0.51, 1.39)	0.5	1.23 (0.81, 1.87)	0.3	1.48 (1, 2.2)	0.05
Local authorities/leaders support IS use	0.94 (0.58, 1.52)	0.8	0.85 (0.57, 1.26)	0.4	0.99 (0.7, 1.39)	0.9

## Supplementary Materials

The following are available online at http://www.mdpi.com/1660-4601/14/7/745/s1, Text S1: Background information titled: The three study regions, Table S1: Independent variables for logistic regression analysis; lists and describes all 16 independent variables and the amount of missing data, Text S2: Methods section titled: Multiple imputations using chained equations; a detailed description about assumptions and methods underlying multiple imputation of missing independent variables, Table S2: Comparison between observations with missing variables and observations that are fully observed; compares observations that have no missing values in any of the variables with observations that have missing observations in one or more of variables, Table S3: Results of multivariable logistic regression analysis of daily IS use, analysis of complete cases and imputed data; comparison of the respective regression model when complete cases versus imputed data is used, Table S4: Results of multivariable logistic regression analysis of LPG stove use, analysis of complete cases and imputed data; comparison of the respective regression model when complete cases versus imputed data is used, Table S5: Results of multivariable logistic regression analysis of traditional cookstove displacement, analysis of complete cases and imputed data; comparison of the respective regression model when complete cases versus imputed data is used, Text S3: Background information titled: Information on institutions/programmes promoting improved stoves in Peru, Table S6: Number of improved solid fuel stoves by region, Table S7: Description of institutions and programmes promoting improved solid fuel stoves; gives detailed information on the institution/stove programme, the stove model, technical specifications, type of fuel and stove certification, Text S4: Background information titled: The national gas programme FISE.

## References

[1] Bonjour S., Adair-Rohani H., Wolf J., Bruce N.G., Mehta S., Prüss-Ustün A., Lahiff M., Rehfuess E.A., Mishra V., Smith K.R. (2013). Solid Fuel Use for Household Cooking: Country and Regional Estimates for 1980–2010. Environ. Health Perspect..

[2] Kurmi O.P., Semple S., Simkhada P., Smith W.C.S., Ayres J.G. (2010). COPD and chronic bronchitis risk of indoor air pollution from solid fuel: A systematic review and meta-analysis. Thorax.

[3] Dherani M., Pope D., Mascarenhas M., Smith K.R., Weber M., Bruce N. (2008). Indoor air pollution from unprocessed solid fuel use and pneumonia risk in children aged under five years: A systematic review and meta-analysis. Bull. World Health Organ..

[4] Uzoigwe J.C., Prum T., Bresnahan E., Garelnabi M. (2013). The Emerging Role of Outdoor and Indoor Air Pollution in Cardiovascular Disease. N. Am. J. Med. Sci..

[5] World Health Organization World Health Organization. Global Health Observatory (GHO) Data. Global Health Observatory (GHO) Data.

[6] Bruce N., Dherani M., Liu R., Hosgood H.D., Sapkota A., Smith K.R., Straif K., Lan Q., Pope D. (2015). Does household use of biomass fuel cause lung cancer? A systematic review and evaluation of the evidence for the GBD 2010 study. Thorax.

[7] Smith K.R., Bruce N., Balakrishnan K., Adair-Rohani H., Balmes J., Chafe Z., Dherani M., Hosgood H.D., Mehta S., Pope D. (2014). Millions dead: How do we know and what does it mean? Methods used in the comparative risk assessment of household air pollution. Annu. Rev. Public Health.

[8] Forouzanfar M.H., Afshin A., Alexander L.T., Anderson H.R., Bhutta Z.A., Biryukov S., Brauer M., Burnett R., Cercy K., Charlson F.J. (2016). Global, regional, and national comparative risk assessment of 79 behavioural, environmental and occupational, and metabolic risks or clusters of risks, 1990–2015: A systematic analysis for the Global Burden of Disease Study 2015. Lancet.

[9] Ruiz-Mercado I., Masera O., Zamora H., Smith K.R. (2011). Adoption and sustained use of improved cookstoves. Energy Policy.

[10] Künzli N. (2011). Commentary: Abating climate change and lung cancer!. Int. J. Epidemiol..

[11] Chafe Z.A., Brauer M., Klimont Z., Van Dingenen R., Mehta S., Rao S., Riahi K., Dentener F., Smith K.R. (2014). Household Cooking with Solid Fuels Contributes to Ambient PM_2.5_ Air Pollution and the Burden of Disease. Environ. Health Perspect..

[12] World Bank (2011). Household Cookstoves, Environment, Health, and Climate Change: A New Look at an Old Problem.

[13] Global Alliance for Clean Cookstoves. Our Mission. http://cleancookstoves.org/about/our-mission/.

[14] United Nations The Sustainable Development Goals Agenda.

[15] Hanna R., Duflo E., Greenstone M. (2012). Up in Smoke: The Influence of Household Behavior on the Long-Run Impact of Improved Cooking Stoves.

[16] Pillarisetti A., Vaswani M., Jack D., Balakrishnan K., Bates M.N., Arora N.K., Smith K.R. (2014). Patterns of stove usage after introduction of an advanced cookstove: The long-term application of household sensors. Environ. Sci. Technol..

[17] Johnson M.A., Chiang R.A. (2015). Quantitative guidance for stove usage and performance to achieve health and environmental targets. Environ. Health Perspect..

[18] World Health Organization (2014). Indoor Air Quality Guidelines: Household Fuel Combustion.

[19] Ruiz-Mercado I., Masera O. (2015). Patterns of Stove Use in the Context of Fuel–Device Stacking: Rationale and Implications. EcoHealth.

[20] Puzzolo E., Stanistreet D., Pope D., Bruce N., Rehfuess E. (2013). Factors Influencing the Largescale Uptake by Households of Cleaner and More Efficient Household Energy Technologies.

[21] Shen G., Lin W., Chen Y., Yue D., Liu Z., Yang C. (2015). Factors influencing the adoption and sustainable use of clean fuels and cookstoves in China—A Chinese literature review. Renew. Sustain. Energy Rev..

[22] Global Alliance for Clean Cookstoves (2011). Igniting Change: A Strategy for Universal Adoption of Clean Cookstoves and Fuels.

[23] Lewis J.J., Pattanayak S.K. (2012). Who adopts improved fuels and cookstoves? A systematic review. Environ. Health Perspect..

[24] Beyene A.D., Koch S.F. (2013). Clean fuel-saving technology adoption in urban Ethiopia. Energy Econ..

[25] Thurber M.C., Warner C., Platt L., Slaski A., Gupta R., Miller G. (2013). To promote adoption of household health technologies, think beyond health. Am. J. Public Health.

[26] Shankar A., Johnson M., Kay E., Pannu R., Beltramo T., Derby E., Harrell S., Davis C., Petach H. (2014). Maximizing the benefits of improved cookstoves: Moving from acquisition to correct and consistent use. Glob. Health Sci. Pract..

[27] Instituto Nacional de Estadística e Informática INEI Estadisticas: Población y Vivienda. http://www.inei.gob.pe/estadisticas/indice-tematico/poblacion-y-vivienda/.

[28] Troncoso K. (2014). A Recipe for Developing Adoption & Impact Indices.

[29] Rheingans R., Anderson J.D., Luyendijk R., Cumming O. (2014). Measuring disparities in sanitation access: Does the measure matter?. Trop. Med. Int. Health.

[30] FISE Fondo de Inclusión Social Energético. http://www.fise.gob.pe/index.html.

[31] Pine K., Edwards R., Masera O., Schilmann A., Marrón-Mares A., Riojas-Rodríguez H. (2011). Adoption and use of improved biomass stoves in Rural Mexico. Energy Sustain. Dev..

[32] Rehfuess E.A., Puzzolo E., Stanistreet D., Pope D., Bruce N. (2014). Enablers and barriers to large-scale uptake of improved solid fuel stoves: A systematic review. Environ. Health Perspect..

[33] Hartinger S.M., Lanata C.F., Hattendorf J., Gil A.I., Verastegui H., Ochoa T., Mäusezahl D. (2011). A community randomised controlled trial evaluating a home-based environmental intervention package of improved stoves, solar water disinfection and kitchen sinks in rural Peru: Rationale, trial design and baseline findings. Contemp. Clin. Trials.

[34] Hartinger S.M., Lanata C.F., Gil A.I., Hattendorf J., Verastegui H., Mäusezahl D. (2012). Combining interventions: Improved chimney stoves, kitchen sinks and solar disinfection of drinking water and kitchen clothes to improve home hygiene in rural Peru. Field Actions Sci. Rep..

[35] Thomas E., Wickramasinghe K., Mendis S., Roberts N., Foster C. (2015). Improved stove interventions to reduce household air pollution in low and middle income countries: A descriptive systematic review. BMC Public Health.

[36] Alam A., Tawale N., Patel A., Dibley M.J., Jadhao S., Raynes-Greenow G. (2016). Household Air Pollution Intervention Implications: Findings from Qualitative Studies and a Field Trial of Clean Cookstoves in Two Rural Villages in India. Int. J. Environ. Res. Public Health.

[37] Rosa G., Majorin F., Boisson S., Barstow C., Johnson M., Kirby M., Ngabo F., Thomas E., Clasen T. (2014). Assessing the Impact of Water Filters and Improved Cook Stoves on Drinking Water Quality and Household Air Pollution: A Randomised Controlled Trial in Rwanda. PLoS ONE.

[38] Wilson D.L., Coyle J., Kirk A., Rosa J., Abbas O., Adam M.I., Gadgil A.J. (2016). Measuring and Increasing Adoption Rates of Cookstoves in a Humanitarian Crisis. Environ. Sci. Technol..

[39] Ruiz-Mercado I., Canuz E., Walker J.L., Smith K.R. (2013). Quantitative metrics of stove adoption using Stove Use Monitors (SUMs). Biomass Bioenergy.

