# Supplementary material for: Adoption of Clean Cookstoves after Improved Solid Fuel Stove Programme Exposure: A Cross-Sectional Study in Three Peruvian Andean Regions
Source: Int J Environ Res Public Health. 2017 Jul 8;14(7):745. doi: 10.3390/ijerph14070745 (PMC5551183; doi:10.3390/ijerph14070745)
Supplement: Supplementary file 1 [file ijerph-14-00745-s001.pdf]

International Journal of
Environmental Research and Public Health

Supplementary Material

Adoption of Clean Cookstoves after Improved Solid Fuel Stove Programme Exposure: A Cross-Sectional Study in Three Peruvian Andean Regions

Jennyfer Wolf, Daniel Mäusezahl, Hector Verastegui and Stella M. Hartinger

Text S1. The Three Study Regions

Cajamarca region is located in the northern highlands of Peru and its capital is Cajamarca. The population is mainly rural (67%) and the major activity of the family head is agriculture (33%) and non-qualified work (31%). 77% of the population uses biomass fuels for cooking. The Cajamarca region currently has over 45,673 certified improved solid fuel stoves installed and 52,000 JUNTOS (National cash transfer programme) beneficiaries that have non-certified improved stove.

La Libertad region is located in the central/northern region of Peru, its capital is Trujillo. It reaches all three Peruvian natural regions (coast, Andes and rainforest). 24% of the population is rural, the main activity of the family head is non-qualified (27%) and construction work (40%). 38% of the population uses biomass fuels for cooking. The La Libertad region has 58,732 certified improved solid fuel stoves installed; of these, GIZ and SEMBRANDO installed around 10,500 and 15,000 respectively. The La Libertad has 59,000 JUNTOS beneficiaries.

Cuzco region is located in the southern region of Peru, its capital is Cuzco. 45% of the population is rural and the main activity of the family head is agriculture (26%), non-qualified work (25%) and commerce (18%). 62% of the population uses biomass fuels for cooking. Cuzco region has 41,904 certified improved solid fuel stoves installed, of these 20,000 have been installed by GIZ and the Ministry of Energy and Mining. Cuzco has 73,500 JUNTOS beneficiaries.

Table S1. Independent variables for logistic regression analyses.

Variable	Description	missing values, n (%)
Region	Categorical variable: Cajamarca, La Libertad, Cuzco	-
Age of reporting female	Categorical variable (ordered): <35, 35–<45, 45–<55, ≥55 [1]	20 (1.9%)
Education of reporting female	Categorical variable (ordered): 0 years, 1–6 years, ≥7 years of schooling [2]	22 (2.1%)
Civil status of reporting female	Binary variable: married/civil partnership vs. single/separated/divorced/widowed	2 (0.2%)
Household size	Binary variable: <5, ≥5 persons	-
Wealth index	Categorical variable (ordered): Quintiles of wealth index	-
Land ownership (where house is built)	Binary variable: yes/no	-
Internet use	Binary variable: any household member uses internet- yes/no	-
Firewood is bought	Binary variable: household buys firewood (vs. collects firewood)- yes/no	-
Stove programme	Categorical variable: IIN, Sembrando, JUNTOS, Municipalidad, NINA, other (various stove programmes that covered less than 5% of the study group)	-
Taking part in the national gas programme	Binary variable: yes/no	-
Year of stove programme participation	Categorical variable (ordered): ≤2008, 2009, 2010, 2011, 2012, 2013	118 (11.4%)
Problems with IS	Binary variable: household encountered problems with IS- yes/no	-
Knowledge of somebody who can build an IS	Binary variable: yes/no	-
IS parts can be obtained in community	Binary variable: yes/no	-
Local authorities/leaders support IS use	Binary variable: yes/no	-

[1] age was categorized so that each group would consist of approximately a quarter of observations, i.e. <35: 24.2%, 35-<45: 28.5%, 45-<55: 24.6% and >55 years: 22.7% (not counting missing values), age was additionally tested as linear variable and adding a quadratic and cubic term to the linear variable; [2] primary school in Peru consists of six years, education was additionally tested as linear variable; IS: improved solid fuel stove

Text S2. Multiple Imputations Using Chained Equations

The missing data pattern was explored and found to be mainly arbitrary, only missingness in the variable of mother`s age was nested in the missingness of mother`s education. These two questions were asked consecutively in the questionnaire.

Four independent variables of our analyses models had missing values and were imputed: age, education and civil status of the reporting female and the calendar year of stove programme participation. Data was imputed with multiple imputations using chained equations in Stata using the ICE command. Twenty imputations were run. All variables from the analyses model plus the three outcomes were used for the imputations of the different variables with missing data. No further auxiliary variables were included in the imputation model.

In our opinion, the missing data mechanism observed in this dataset is most likely missing-at-random (MAR). Table A2 compares demographics and further variables of observations with and without missing values. It shows that from the 138 observations with missing values a much higher percentage lived in Cuzco (62% vs. 27%) compared to the remainder with no missing values. In a logistic

regression, examining an indicator of missingness in the "year of stove programme" variable, we observed associations with region and stove programme (no associations with any other variable used in the analyses models). An increase in missing values might for example be due to a different group of fieldworkers in the respective region. Furthermore there had been circumstances in Cuzco which partly impeded data collection such as a strike of ministry of health personnel. The other two missing data mechanisms, missing-completely-at-random (MCAR) and missing-not-at-random (MNAR) might also be possible: The variable with by far the most missing values (calendar year when cookstove programme was conducted) was a sub-question to the question in which stove programme the household had participated. This question appeared towards the end of a rather long questionnaire and the sub-question might therefore have been occasionally skipped or forgotten (which would make the missing data mechanism MCAR). If missingness in the variable calendar year of stove programme participation was related to the values of the variable itself (i.e. missing value when the cookstove programme had been conducted a long time age and the reporting female did therefore not remember), then the missing data mechanism would be MNAR in which case multiple imputation might lead to biased estimates [1]. We believe that the latter is unlikely as the observations would have rather been coded as "not known".

We compare the results of our three analyses models from complete case analysis and analysis of multiply imputed data in Tables A3-A5. We additionally tested the sensitivity of our results to the number of imputations as well as the specific imputations used. We did not find any relevant change in the effect estimates for the three models running only ten imputations or using only a specific subset of the imputations. For one stove programme we knew that it had been conducted in only one specific year. This gave us the possibilities to check the imputations to a certain degree. In almost all missing cases in this stove programme the respective year was imputed.

Table S2. Comparison between observations with missing variables and observations that are fully observed.

	**missing, N = 138**	**non-missing, N = 895**
Region		
Cajamarca	32 (23.2)	312 (34.9)
Cuzco	86 (62.2)	245 (27.4)
La Libertad	20 (14.5)	338 (37.8)
Age of reporting female		
<35	23 (16.7)	222 (24.8)
35–<45	40 (29.0)	249 (27.8)
45–<55	26 (18.8)	223 (24.9)
≥55	29 (21.0)	201 (22.5)
Education of reporting female (years of schooling)		
0	18 (13.0)	115 (12.9)
1–6	73 (52.9)	628 (70.2)
≥7	25 (18.1)	152 (17.0)
Civil status of reporting female, married/civil partnership vs. single/separated/divorced/widowed	114 (82.6)	762 (85.1)
Household size, ≥5 persons	56 (40.6)	417 (46.6)
Wealth index		
1. Quintile (lowest)	25 (18.1)	182 (20.3)
2. Quintile	27 (19.6)	185 (20.7)
3. Quintile	27 (19.6)	188 (21.0)
4. Quintile	29 (21.0)	168 (18.8)
5. Quintile (highest)	30 (21.7)	172 (19.2)
Land ownership (where house is built)	126 (91.3)	789 (88.2)
Internet use	46 (33.3)	295 (33.0)
Firewood is bought	48 (34.8)	314 (35.1)
Stove programme		
IIN	5 (3.6)	142 (15.9)
Sembrando	7 (5.1)	184 (20.6)
Juntos	17 (12.3)	166 (18.6)
Municipalidad	23 (16.7)	95 (10.6)
Nina	45 (32.6)	192 (21.5)
others (various, <5%)	41 (29.7)	116 (13.0)
Year of stove programme participation		
≤2008	-	197 (22.0)
2009	6 (4.4)	155 (17.3)
2010	7 (5.1)	186 (20.8)
2011	5 (3.6)	204 (22.8)
2012	2 (1.5)	103 (11.5)
2013	-	50 (5.6)
Problems with IS	29 (21.0)	309 (34.5)
Knowledge of somebody who can build IS	45 (32.6)	287 (32.1)
IS parts can be obtained in community	19 (13.8)	155 (17.3)
Local authorities/leaders support IS use	38 (27.5)	397 (44.4)

IS: improved solid fuel stove.

Table S3. Results of multivariable logistic regression analysis of daily IS use, analysis of complete cases and imputed data.

**Daily IS use**	**Complete cases (n = 895)**			**Imputed (n = 1,033)**		
	OR	**(95% CI)**	*p*	**OR**	**(95% CI)**	*p*
Region			0.3			0.3
Cajamarca	1			1		
La Libertad	1.18	(0.37, 3.83)		1.27	(0.44, 3.68)	
Cuzco	3.73	(0.77, 18.17)		2.46	(0.83, 7.29)	
Age of reporting female			0.3			0.4
<35	1			1		
35–<45	0.68	(0.39, 1.18)		0.75	(0.44, 1.28)	
45–<55	1.19	(0.63, 2.26)		1.26	(0.68, 2.32)	
≥55	0.94	(0.47, 1.91)		0.96	(0.49, 1.87)	
Education of reporting female (years of schooling)			0.02			0.02
0	1			1		
1–6	0.84	(0.41, 1.73)		0.90	(0.46, 1.76)	
≥7	0.40	(0.17, 0.94)		0.44	(0.20, 0.99)	
Civil status of reporting female, married/civil partnership vs. single/separated/divorced/widowed	1.73	(1.02, 2.94)	0.04	1.75	(1.06, 2.88)	0.03
Household size, ≥5 persons	1.30	(0.83, 2.04)	0.3	1.25	(0.82, 1.91)	0.3
Wealth index			0.9			0.9
1. Quintile (lowest)	1			1		
2. Quintile	0.98	(0.52, 1.85)		1.05	(0.57, 1.93)	
3. Quintile	0.79	(0.42, 1.49)		0.77	(0.43, 1.4)	
4. Quintile	0.83	(0.42, 1.65)		0.93	(0.48, 1.78)	
5. Quintile (highest)	1.05	(0.51, 2.18)		0.91	(0.46, 1.79)	
Land ownership (where house is built)	0.99	(0.52, 1.86)	1	1.06	(0.58, 1.93)	0.9
Internet use	1.33	(0.8, 2.2)	0.3	1.36	(0.85, 2.18)	0.2
Firewood is bought	0.77	(0.49, 1.22)	0.3	0.80	(0.52, 1.23)	0.3
Stove programme			0.08			0.2
IIN	1			1		
Sembrando	2.42	(0.77, 7.62)		2.32	(0.74, 7.25)	
Juntos	2.57	(0.72, 9.19)		2.37	(0.68, 8.27)	
Municipalidad	2.93	(0.64, 13.47)		3.21	(0.75, 13.76)	
Nina	0.77	(0.1, 5.77)		1.42	(0.28, 7.32)	
others (various, <5%)	5.08	(1.38, 18.75)		3.91	(1.11, 13.78)	
Year of stove programme participation			0.2			0.2
≤2008	1			1		
2009	0.45	(0.11, 1.8)		0.48	(0.13, 1.86)	
2010	0.32	(0.08, 1.28)		0.33	(0.09, 1.24)	
2011	0.47	(0.12, 1.94)		0.41	(0.11, 1.61)	
2012	0.85	(0.2, 3.66)		0.77	(0.19, 3.22)	
2013	0.28	(0.06, 1.28)		0.31	(0.07, 1.38)	
Problems with IS	0.55	(0.36, 0.84)	0.01	0.62	(0.41, 0.93)	0.02
Knowledge of somebody who can build IS	1.80	(1.09, 2.97)	0.02	2.01	(1.24, 3.27)	0.01
IS parts can be obtained in community	0.82	(0.48, 1.4)	0.5	0.84	(0.51, 1.39)	0.5
Local authorities/leaders support IS use	1.02	(0.61, 1.71)	0.9	0.94	(0.58, 1.52)	0.8

IS: improved solid fuel stove.

Table S4. Results of multivariable logistic regression analysis of LPG stove use, analysis of complete cases and imputed data.

**LPG stove use**	**Complete cases (n = 728)**			**Imputed (n = 844)**		
	**OR**	**(95% CI)**	***p***	**OR**	**(95% CI)**	***p***
Region			0.3			0.3
Cajamarca	1			1		
La Libertad	1.29	(0.48, 3.5)		1.19	(0.48, 2.94)	
Cuzco	2.00	(0.85, 4.71)		1.71	(0.87, 3.36)	
Age of reporting female			0.7			1
<35	1			1		
35–<45	0.76	(0.46, 1.25)		0.89	(0.56, 1.42)	
45–<55	0.82	(0.49, 1.4)		0.94	(0.57, 1.54)	
≥55	0.89	(0.49, 1.64)		0.93	(0.53, 1.63)	
Education of reporting female (years of schooling)			0.1			0.02
0	1			1		
1–6	1.80	(1, 3.24)		2.09	(1.22, 3.6)	
>7	1.99	(0.95, 4.18)		2.47	(1.26, 4.87)	
Civil status of reporting female, married/civil partnership vs. single/separated/divorced/widowed	0.88	(0.53, 1.46)	0.6	0.97	(0.62, 1.54)	0.9
Household size, ≥5 persons	0.83	(0.57, 1.23)	0.4	0.80	(0.56, 1.13)	0.2
Wealth index			<0.0001			<0.0001
1. Quintile (lowest)	1			1		
2. Quintile	2.31	(1.29, 4.16)		2.41	(1.4, 4.15)	
3. Quintile	2.06	(1.14, 3.74)		2.03	(1.17, 3.51)	
4. Quintile	5.08	(2.81, 9.21)		5.05	(2.92, 8.74)	
5. Quintile (highest)	12.71	(6.69, 24.15)		10.17	(5.67, 18.22)	
Land ownership (where house is built)	0.95	(0.54, 1.66)	0.9	0.97	(0.57, 1.65)	0.9
Internet use	1.47	(0.99, 2.2)	0.06	1.28	(0.89, 1.83)	0.2
Firewood is bought	1.22	(0.83, 1.8)	0.3	1.09	(0.76, 1.55)	0.6
Stove programme		0.2				0.07
IIN	1			1		
Sembrando	1.05	(0.43, 2.55)		1.04	(0.44, 2.47)	
Juntos	2.31	(0.84, 6.35)		2.61	(0.99, 6.9)	
Municipalidad	0.98	(0.32, 3)		1.13	(0.4, 3.21)	
Nina	1.21	(0.35, 4.2)		1.43	(0.47, 4.34)	
others (various, <5%)	1.37	(0.53, 3.51)		1.39	(0.57, 3.43)	
Year of stove programme participation			0.05			0.1
≤2008	1			1		
2009	0.47	(0.17, 1.33)		0.54	(0.2, 1.46)	
2010	0.87	(0.32, 2.34)		0.90	(0.36, 2.26)	
2011	0.81	(0.29, 2.21)		0.87	(0.34, 2.25)	
2012	1.70	(0.62, 4.64)		1.61	(0.62, 4.16)	
2013	0.89	(0.29, 2.76)		0.98	(0.33, 2.88)	
Problems with IS	1.13	(0.78, 1.66)	0.5	1.13	(0.8, 1.6)	0.5
Knowledge of somebody who can build IS	0.61	(0.41, 0.92)	0.02	0.68	(0.47, 0.98)	0.04
IS parts can be obtained in community	1.32	(0.84, 2.07)	0.2	1.23	(0.81, 1.87)	0.3
local authorities/leaders support IS use	0.82	(0.53, 1.27)	0.4	0.85	(0.57, 1.26)	0.4

IS: improved solid fuel stove, LPG: liquefied petroleum gas.

Table S5. Results of multivariable logistic regression analysis of traditional cookstove displacement, analysis of complete cases and imputed data.

**Traditional cookstove displacement**	**Complete cases (n = 895)**			**Imputed (n = 1,033)**		
	**OR**	**(95% CI)**	***p***	**OR**	**(95% CI)**	***p***
Region			0.0004			<0.0001
Cajamarca	1			1		
La Libertad	2.80	(1.3, 6.06)		2.42	(1.19, 4.93)	
Cuzco	4.74	(2.01, 11.21)		4.48	(2.28, 8.79)	
Age of reporting female			0.0009			0.002
<35	1			1		
35–<45	0.53	(0.34, 0.82)		0.59	(0.39, 0.89)	
45–<55	0.39	(0.25, 0.63)		0.43	(0.28, 0.67)	
≥55	0.44	(0.26, 0.75)		0.49	(0.3, 0.81)	
Education of reporting female (years of schooling)			0.3			0.2
0	1			1		
1–6	1.13	(0.7, 1.83)		1.39	(0.89, 2.17)	
>7	0.81	(0.43, 1.52)		1.03	(0.57, 1.84)	
Civil status of reporting female, married/civil partnership vs. single/separated/divorced/widowed	1.07	(0.69, 1.66)	0.8	0.98	(0.65, 1.47)	0.9
Household size, ≥5 persons	0.63	(0.45, 0.88)	0.006	0.64	(0.47, 0.88)	0.005
Wealth index			0.003			0.001
1. Quintile (lowest)	1			1		
2. Quintile	1.45	(0.92, 2.28)		1.40	(0.92, 2.14)	
3. Quintile	1.69	(1.06, 2.7)		1.81	(1.17, 2.8)	
4. Quintile	1.22	(0.75, 1.98)		1.27	(0.81, 1.99)	
5. Quintile (highest)	2.77	(1.61, 4.77)		2.74	(1.65, 4.54)	
Land ownership (where house is built)	0.75		0.3	0.77	(0.47, 1.26)	0.3
Internet use	1.04		0.8	1.07	(0.76, 1.5)	0.7
Firewood is bought	1.39		0.07	1.28	(0.92, 1.77)	0.1
Stove programme			0.1			0.1
IIN	1			1		
Sembrando	1.47	(0.72, 3)		1.48	(0.73, 2.98)	
Juntos	0.79	(0.35, 1.79)		0.80	(0.36, 1.78)	
Municipalidad	0.63	(0.25, 1.62)		0.66	(0.27, 1.64)	
Nina	0.52	(0.17, 1.61)		0.51	(0.19, 1.39)	
others (various, <5%)	0.96	(0.46, 2)		0.93	(0.45, 1.93)	
Year of stove programme participation			0.1			0.2
≤2008	1			1		
2009	0.86	(0.38, 1.95)		0.96	(0.43, 2.14)	
2010	0.86	(0.38, 1.93)		1.00	(0.46, 2.17)	
2011	1.15	(0.5, 2.69)		1.24	(0.53, 2.91)	
2012	1.27	(0.55, 2.95)		1.42	(0.62, 3.25)	
2013	3.47	(1.24, 9.67)		3.23	(1.18, 8.84)	
Problems with IS	0.61	(0.44, 0.83)	0.002	0.65	(0.48, 0.88)	0.005
Knowledge of somebody who can build IS	0.94	(0.67, 1.32)	0.7	1.02	(0.74, 1.41)	0.9
IS parts can be obtained in community	1.53	(1, 2.33)	0.05	1.48	(1, 2.2)	0.05
local authorities/leaders support IS use	0.96	(0.66, 1.39)	0.8	0.99	(0.7, 1.39)	0.9

IS: improved solid fuel stove.

Text S3. Information on Institutions/Programmes Promoting Improved Stoves in Peru

In 2009, a legislative change enabled that part of the government's national mining revenues could be invested in the implementation of improved stoves. These endeavours were part of the initiative "Half million improved stoves for a smokeless Peru" campaign (2009–2011). More than 287 thousand improved solid fuel stoves were installed nationwide. The private sector (NGOs and private companies) played a major role and was responsible for installing 43% of improved stoves. In 2012, two additional programmes were created: (i) the NINA programme, with the objective of substituting kerosene and other unimproved stoves (such as open fire) with liquefied petroleum gas (LPG) and improved solid fuel stoves; and (ii) the Social Inclusion Energy Fund (FISE). The last estimate of the number of installed improved stoves nationwide (2014) was 301'088. The regions with the highest numbers of improved stoves were Cajamarca, La Libertad and Cusco (Table S6).

Table S6. Number of improved solid fuel stoves by region.

Peruvian regions	number of stoves installed until 2014
AMAZONAS	5,182
ANCASH	1,687
APURIMAC	18,179
AREQUIPA	14,404
AYACUCHO	17,695
CAJAMARCA	45,673
CUZCO	41,904
HUANUCO	6,086
HUANCAVELICA	34,770
ICA	624
JUNIN	2,168
LA LIBERTAD	58,732
LAMBAYEQUE	1,949
LIMA	3,183
LORETO	3,509
MOQUEGUA	4,073
PASCO	4,983
PIURA	20,652
PUNO	1,154
SAN MARTIN	4,159
TACNA	9,802
UCAYALI	520

Source: Grupo Tematico Energia para Cocinar. Mejorando la Calidad de Vida. http://www.cocinasmejoradasperu.org.pe/nosotros.html.

Table S7. Description of institutions and programmes promoting improved solid fuel stoves.

Institution/Stove programme	Stove Model	Technical Specification of the stove	Type of Programme	Type of Fuel	Certified
IIN; Instituto de Investigación Nutricional (www.iin.sld.pe/)	OPTIMA	Chimney, 3 furnaces, combustion chamber made of local materials (clay and mud)	The IIN is a non-profit Peruvian institution that works in several research projects linked to nutrition, health and the environment.	Biomass (wood and dung)	Yes, 2015
Programa Sembrando (www.sembrando.org.pe/)	Incahuasi Sembrando 1 y 2	Chimney, 2 or 3 furnaces with cement slab, closed combustion chamber made of refractory clay	As one part of their social and economic development programme, Sembrando promotes improved stoves.	Biomass (wood and dung)	Yes, 2009 & 2012
JUNTOS; Programa Nacional de Apoyo a los más Pobres (www.juntos.gob.pe/)	Locally made stoves	Chimney, 2 or 3 furnaces, combustion chamber made of local materials (clay and mud)	Governmental conditional cash transfer programme implemented in 2005. The programme transfers the equivalent of about US$30 monthly to poor and extremely poor families. The main conditions for families to participate are that all children < 5 must attend to the health centre for growth monitoring, all must attend school, and all pregnant women must attend regular check-ups. In addition, household had to have improved stoves installed (this requirement was later removed) and latrines.	Biomass (wood and dung)	
Municipalidad	Incahuasi GIZ or local model	Chimney, 2 or 3 furnaces with cement slab, closed combustion chamber made of refractory clay	Local programme varied between municipalities	Biomass (wood and dung)	
Ministerio de Energía y Minas – MINEM, Project NINA, (www.minem.gob.pe/descripcion.php?idSector=5&idTitular=3351)	Incahuasi Sembrando or GIZ	Chimney, 2 or 3 furnaces with cement slab, closed combustion chamber made of refractory clay	Governmental programme by the Ministry of Energy and Mining to improve energy efficiency. The programme ((302-2012-MEM/DM), was created to substitute kerosene and other contaminating stoves with LPG and improved stoves	Biomass (wood and dung)	Yes, 2010, 2012 & 2014

Text S4. The National Gas Programme FISE

The Social Inclusion Energy Fund (FISE) was created in 2012 under the Law N° 29852 of the Ministry of Energy of Mining. FISE, constituted as a social inclusion policy mechanism of the Peruvian government, has the aim of expanding the energy sector to poor and extremely poor parts of the

country, promoting access to energy. It promotes the access of LPG, by providing subsidies to purchase LPG cylinders (monthly discount vouchers of ~US$5.5). The administration of FISE (until 2017) is under the Supervisory Board for Investment in Energy and Mines (Osinergmin). By 2015, more than 1.3 million families nationwide had received an LPG stove and participated in the FISE programme, mostly located in the regions of Lima, Puno, Junín and Cuzco.

References

1. Sterne, J.A.; White, I.R.; Carlin, J.B.; Spratt, M.; Royston, P.; Kenward, M.G.; Wood, A.M.; Carpenter, J.R.. Multiple imputation for missing data in epidemiological and clinical research: Potential and pitfalls. *BMJ* **2009**, *338*, b2393.
